# Editorial

**Published:** 1904-09-15

**Authors:** 


### EDITORIAL.

THE FOURTH INTERNATIONAL DENTAL CONGRESS NOW HISTORY.—The last session of what was in some respects the most important dental convention ever held, took place September the second in the Coliseum at St. Louis, and partook largely of a congratulatory love feast. The opening of the Congress was expected to be more or less turbulent, because of the dissatisfaction as to the methods of the committee on organization in electing the presiding officials. The contest, however, was very brief and attended with no acrimonious discussion. The vote was decisive and accepted gallantly by all contestants. The opening session consisted of introduction of the representatives of the foreign nations, and was an exceedingly interesting part of the program. The contributions of foreign dentists were many and of great value. It would be difficult, indeed, to estimate the value of this great contribution to dental science and art. In fact, no one can form any just conception until he has had a chance to review the published proceedings, as the papers were read simultaneously in ten different sections. It kept one busy following up the papers he was most interested in hearing, and it sometimes happened that two papers were being read at the same instant that he wanted very much to hear. The first prize paper was read by Dr. W. D. Miller, in the general section, as were other excellent papers. The clinics were probably the best ever presented at any meeting, and there were many of them. There was a great variety of subjects, from filling teeth to surgical operations for cleft palate, etc., and great interest was taken in them. The facilities for the clinics were very good. The room was commodious and well lighted. In fact, the clinical facilities were far superior to the accomodations for the literary work. The building was exceedingly noisy and its acoustics almost unbearable. In two of the section rooms there was no other means than a skylight for ventilation. There were more than 1600 dentists registered. Many attended who did not register. There were nearly 150 from foreign countries. Nearly every country where dentistry is practiced sent a delegate. France and Germany were well represented, England by two delegates only. The task of reporting the proceedings was well handled by the *Dental Cosmos*, with ten short-hand reporters. Holding the meeting in the same city with the great World's Fair did not tend to a good attendance at all the meetings, as many delegates divided their time between these two great attractions. The weather was especially comfortable, with the single exception of the night of the great banquet, when it was too hot to be at all comfortable. The dentists of St. Louis and the local committee deserve much praise for the many courtesies extended to those attending the Congress. It was a great meeting, and to those who bore the task of organization and conduct, too much praise and congratulation may not be given.

—✦—

THE NATIONAL BOARD OF EXAMINERS.—This organization met at St. Louis the week before the International Congress, and spent most of its time in discussing the educational situation. Much dissatisfaction was expressed at the return of the colleges to a three-year course. The whole subject was convassed in the president's address and the report of the committee on colleges. A two-days' discussion of the situation resulted in the adoption by this body of a standard of education which it deems essential for recognition. This standard is twenty-eight weeks of instruction and a high-school graduation for admission, to take effect with the session beginning in 1905. Just what effect this will have on the colleges remains to be seen. No college in the United States can, at present, qualify under this standard. Some schools have twenty-seven months of instruction and the high-school requirement for admission. It means a four-year course of seven months. The sentiment seemed to be that there was no probability that the dental colleges would ever again attempt an advance, and that the establishment of a standard should come from some source other than the Faculties Association. The examiners have established standards of reputability before but always in harmony with action by the Faculties Association. It remains to be seen what the result of this action will be. There can be no doubt that should the Examiners Association adopt a reasonable standard, and be able to put it into practice in a considerable majority of the States, that the colleges would very soon come to it. It would also be a powerful influence in shaping future legislation to the extent of producing the long desired uniformity in legislation; thus furnishing a practicable basis for inter-state comity. It would do another good thing and that is to take away from the Faculties Association all excuse for spending the time of its session in endless legislation, and give it a chance to take up the functions for which it was organized. The National Examiners Association is an organization of intelligent dentists, representing the people and the laws made for their protection, unprejudiced by association of any sort with the business of educating dentists and ought, therefore, to be peculiarly qualified to pass on the matter of the quantity as well as the quality of training needed to entitle one to offer his services to the public with legal endorsement. Let us hope that we are at last to have a solution of this vexing problem that will be final and satisfactory.

—✦—

THE INTERNATIONAL DENTAL FEDERATION.—This organization held a two-days' session on Friday and Saturday before the Congress opened. Its purpose is to establish an International Curriculum of education. The convention was well attended by representatives from several foreign countries, England being conspicuously absent, but she evidently does not desire to compromise her ideals for the sake of uniformity. Much of the first session was taken up with formal introductions and felicitous exchanges of greetings, which reminded one of diplomatic procedures, and it seemed to crop out in all subsequent proceedings to a considerable extent.

In the discussions it seemed that ways and means should not wholly rest upon scientific pedagogy, but that diplomacy should be utilized as a powerful ally in accomplishing international uniformity. It is probably true that diplomacy shall eventually get the credit for the success desired. It seems proper in a meeting of this kind that the subject should be studied wholly from the scientific standpoint. It does not necessarily follow that a nation's peculiar methods are the only ones that can be used successfully in that country. It may mean that they know no other. The general nature of man is the same everywhere, and the most scientific methods will succeed best wherever used.

